# An Integrated Theatre Production for School Nutrition Promotion Program

**DOI:** 10.3390/children5030035

**Published:** 2018-03-02

**Authors:** Robert Bush, Sandra Capra, Selina Box, David McCallum, Stephanie Khalil, Remo Ostini

**Affiliations:** 1 School of Public Health, The University of Queensland, Herston 4006, Australia; r.bush@uq.edu.au; 2 School of Human Movement and Nutrition Sciences, The University of Queensland, St. Lucia 4072, Australia; s.capra@uq.edu.au; 3 Eduhealth Plus, Ipswich 4001, Australia; admin@eatsmartbactive.com.au (S.B.); admin@eduhealthplus.com.au (D.M.); 4 School of Public Health, The University of Queensland, Herston 4006, Australia; Stephanie.khalil@uqconnect.edu.au; 5 Rural Clinical School Research Centre, The University of Queensland, Toowoomba 4350, Australia

**Keywords:** theatre production, integrated school-based programs, childhood nutrition, healthy food behaviour, super-settings

## Abstract

In the context of stubbornly high childhood obesity rates, health promotion activities in schools provide a potential avenue to improve children’s nutritional behaviours. Theatre production has a rich history as a health behaviour promotion strategy but lacks sound, outcome-based evaluation. This study evaluated the effect of an integrated, two-part, place-based theatre performance program with 212 students in five schools in a regional urban and semi-rural area. The program included a theatre performance and a healthy eating competition. A brief survey assessed student healthy eating knowledge and attitudes at three time points. Nutrition behaviour was measured by scoring the contents of children’s lunch boxes before, during and up to six weeks after the intervention. Statistical analysis tested change over time on five variables (Knowledge, Attitude, Sometimes foods, Everyday foods, Overall lunch box score). Results showed that both components of the integrated program improved nutrition knowledge and that the theatre performance improved children’s healthy eating attitudes. All three lunch box scores peaked after the integrated program and remained significantly higher than baseline at 4–6 weeks follow-up. Interaction effects were identified for school catchment area on four of the five dependent variables. Evaluation of this integrated theatre production program indicates the potential benefit of taking a “super-setting” approach. It demonstrates an effect from students taking home information they had learned and incorporating it into lunch box preparation. It also showed consistent effects for school geographical catchment. This study suggests that, with careful, theory-based design, theatre productions in schools can improve student nutritional activities.

## 1. Introduction

Theatre production as a health promotion strategy has a considerable history and descriptions of its application abound [1]. However, confidence in its efficacy has been hampered by a lack of careful and systematic analysis of how theatre production may work to enhance health promotion, and a limited amount of evidence for soundly-based outcomes [2]. Here we address these two short-comings in a study of the use of theatre production as part of an integrated package of interventions designed to help change nutrition knowledge, attitudes and behaviours of primary school children in a region of Queensland, Australia. 

Children’s eating habits are increasingly the concern of international agencies and national governments as the impacts of rising obesity become evident [3,4]. Australian evidence for poor nutrition among children, and the intractable nature of reducing the incidence of obesity, is demonstrated from the findings of two Australian national surveys over time. In 2007, the second national Australian survey of nutrition and physical activity in children found high levels of non-observance for intakes of vegetables (too few), saturated fat and sugar (too much) for all children, and low fruit and dairy food intakes for children over 9 years of age [5]. The 2011–2012 National Nutrition Survey suggests that no apparent improvements had been achieved for these indicators [6]. Children’s self-reports showed that less than 7% were consuming the target amount of vegetables, and that 40% of their energy intake came from “discretionary” or less desirable foods, with 60% from the preferred “foundation” or basic foods such as cereal foods, fruit, vegetables meats and dairy foods.

Schools have long been seen to be a potentially effective setting for health promotion to address such intractable problems, as is supported by the World Health Organization’s (WHO’s) Health Promoting School approach [7]. Since the early 1990s Australian policy has likewise encouraged an inter-sectoral approach, with engagement of schools, the community and government to achieve the goals of better nutrition and the reduction of chronic disease [8]. Recently there have been suggestions that using “super-settings” may be more effective than a focus on the school setting alone [9]. In this approach the school is seen as part of a wider context, including aspects of the wider community in which it is situated. Such an approach may co-opt greater levels of resourcing, help to change wider risk and protective factors and support sustainable change through normative environmental shifts. One practical way to understand the “super-setting” beyond the physical boundaries of the school, for example, is to include the family setting and in particular the practice of preparing food lunch boxes at home for consumption at school. This connection between school and home creates a tangible link to demonstrate what is being learned at school. It also leads to changes in food preparation at home.

An integrated approach like this to a school place-based approach requires a sound theoretical underpinning as well as description of how each of the components of an intervention are expected to influence a change in children’s food knowledge, attitude and behaviour [2]. The key components in the current intervention were a theatre performance at school followed by a lunch box competitive challenge. This provides a tangible link between the school and the home through changing the lunch box contents to demonstrate improved alignment with healthy food choices. In this way enhanced learning about healthy foods can be turned into home-based actions to change the children’s daily eating habits.

The theoretical basis of this integrated place-based approach lies in Social Cognitive Theory [10] which outlines how children learn through experience in person-environment interactions to acquire certain behavioural patterns. Here we add an important second theoretical base by including more recent advances in Obesogenic Environment Theory [11,12] which seeks to identify factors in the environment that act as risk and protective factors re-enforcing opportunities and norms of eating behaviour. This is especially significant given that children’s food availability is not usually under their direct control but that of parents and other providers. Indeed the eating behaviour of parents has a major influence on children’s eating habits [13]. If theatre performance is to have health promoting outcomes it needs to be based on a realistic assessment of children’s eating environments and the barriers to change [1]. The theatre performance also needs to match the child’s social, cognitive and emotional development. Reviews of theatre performance generally also recognize passive theatre observation by children is not enough to evoke change in behaviour and that discussion, activity and opportunity to change behaviour need to be incorporated [2]. Studies that do not involve these additional components have found changes to knowledge and attitude but not necessarily in behaviour [14].

As an addition to classroom curriculum-based healthy eating approaches, theatre production seeks to engage through memorable and iconic performance often involving music and acting designed to engage both cognitive and emotional responses in children [15]. As such a key value of theatre is its potential to engage and motivate.

Following up performance by integrating a lunch box challenge incorporates elements of competition and is based on positive finding of the use of competition to motivate children to increase physical activity [15,16,17]. Organizing and maintaining a lunch box competition through school requires school leadership and whole school commitment well beyond having an external provider attend to provide a special theatre performance.

While this school-based leadership and commitment fosters competition at the individual level and engages the children’s home situations, it limits the “super-setting” approach to personalized behavioural change. To also encourage a change in the wider school environment a regional school initiative sought to support wider normative change, schools within the region were given the opportunity to join in a regional best school performance award, organized through the theatre troupe to provide continuity. The data collected in schools about changes to lunch box healthier foods were submitted to a regional challenge completion and a regional school prize award annually.

The aim of this study therefore was to evaluate whether an intervention comprised of a live theatre production followed by an extended healthy eating competition could be an effective strategy for achieving positive behaviour change, using a pre and post test design.

## 2. Materials and Methods

### 2.1. Theatre Performance and Lunch Box Challenge

**Figure 1:**
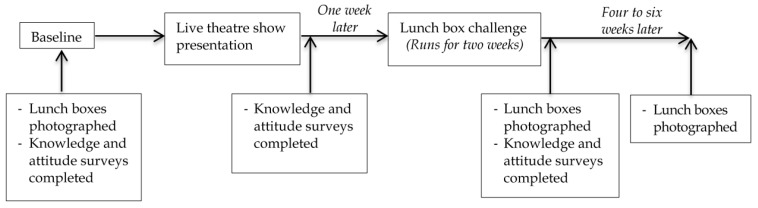
Schematic of study components and timing.

Using available evidence and through consultation with educational and health promotion experts, a children’s entertainment group, The Boogie Woogies Superhero Band (Grande Musical Promotions, Ipswich Australia), developed an integrated package of interventions designed to positively influence primary school children’s food knowledge, attitudes and behaviours. The components of the program were accredited with government and non-government education authorities for use in schools in Queensland, Australia. The program included a 60-min live theatre component addressing nutrition content areas, supported by a lunch box challenge activity focusing on the inclusion of positive foods. Figure 1 shows the components of the study including program components, study measures and the timing of the delivery of each component. The theatre production was delivered on site at each school, using the school hall facilities.

The lunch box challenge measured the content of lunch boxes before and after exposure to the theatre production in children from classes, selected by the principals as not having recently completed a Health/Food and Nutrition unit in their educational curriculum prior to the challenge. The challenge began at the start of the week following the theatre production and continued for 10 consecutive school days. The content of the lunch boxes were measured again four to six weeks after delivery of the theatre production. Lunch boxes were photographed and scored by four researchers one week prior to implementation (baseline) and then at weeks 3 and 7–10. The program was designed to be appropriate for primary school children’s cognitive, emotional and social stage of development. Known as Eat Smart B Active, Healthiest School Awards (Grande Musical Promotions, Ipswich, Australia) the same program was delivered to each child in the study following a set protocol agreed to by school authorities. The program was designed to be practical and to use repetition and practice to reinforce learning. The aim of the program was to teach children about the desirable food groups with the longer term aim of reducing the intake of less desirable foods and challenging them to change their lunches. Families were engaged via newsletters and information sheets which outlined the desirable (Everyday foods) and less desirable foods (Sometimes foods). Success in the lunch box challenge competition required the information that was imparted by the theatre performance to be incorporated into lunch preparation practices in children’s homes.

### 2.2. Intervention Region and Sampling

The location was a regional urban and semi-rural area within South East Queensland Australia. A total of 323 primary schools were located in the region (179 government funded and 144 non-government funded) with the majority of these in the metropolitan area [18]. All socio-economic ranges are included in this group of schools.

A pragmatic design was used with 18 schools approached for potential program participation, all in a geographical radius of 10 km of the regional urban centre. This included outer suburban areas of the metropolitan region on the eastern side and semi-rural sites on the western side. Participation of the program was dependent on the self-selection of the schools. 

Five of the initial 18 schools that were approached declined to participate. The Eat Smart B Active | Healthiest School Awards program was therefore delivered to 13 schools. Of the 13 participating schools, five schools, representing a range of socioeconomic status as well as different student catchment area strategies (local or regional), were purposefully selected to take part in the study of program effects that is reported here. No schools that were approached to take part in the study declined to participate. One of the five study schools had participated in the program in the previous year, while the program was under development and refinement. While the integrated program was delivered to the entire school community in each case, principals at each of the study schools selected two classrooms for inclusion in the study. Children in grades 2 to 5 were eligible to participate in the study as this was the target age range for the intervention (7–11 years) and principals at each school selected an older and a younger grade for study inclusion. This study was approved by the University of Queensland Behavioural and Social Sciences Ethical Review Committee, Approval number 2014001104.

### 2.3. Measures

Students completed a specifically designed brief survey form before the start of intervention activities, following the first activity (theatre performance) and again at the end of the two-week lunch box challenge competition. The survey was reviewed for construct and content validity by a researcher not associated with the study in the development phase of the program prior to its implementation. The final survey contained a 10-item measure of knowledge about healthy eating (for example, listing the five “everyday” food groups, ticking facts about salt and sugar) and a 6-item measure of attitudes towards healthy eating, using 3 point scales of no, unsure, and yes. Correct responses on the knowledge scale were summed for each student to produce a knowledge score that could range from 0 to 30. The number of positive attitudes endorsed by students on the attitude scale was summed to produce an attitude score that could range from 0 to 6.

**Figure 2:**
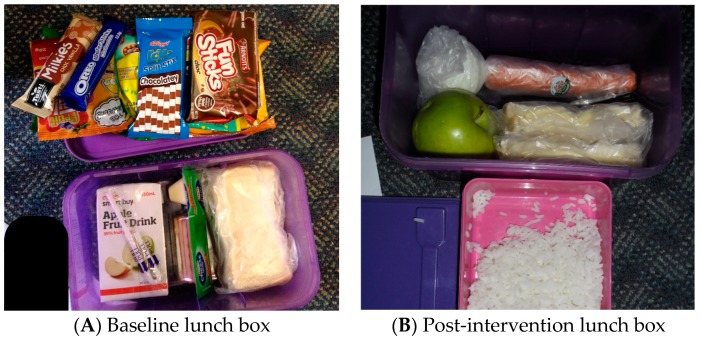
Two lunch box photographs (Scoring (**A**): Grain = 1; Fruit = 0; Vegetables = 0; Dairy = 1; Protein = 0; Sometimes Food = 8; Scoring (**B**): Grain = 2; Fruit = 1; Vegetables = 1; Dairy = 1; Protein = 1; Sometimes Food = 0).

Nutrition behaviour was measured by scoring the content of children’s lunch boxes (1 point for each of the five ‘everyday’ food groups packed in lunch box—i.e., fruit, vegetables, grains, dairy and protein; and 1 point deducted for each ‘sometimes’ food—i.e., packaged food high in sugar, salt or fat). Children’s nutritional behaviour change was measured by photographing and scoring children’s lunch box content at baseline; during the rewards challenge intervention; and four–six weeks after the intervention. The baseline and four–six weeks follow-up measures of lunch box content were conducted without prior student knowledge that they would be occurring. Scoring of lunch box contents from photographs followed a pre-defined protocol and provided an objective way to measure nutritional behaviour that built on previous research by a study author using photographic evidence of meals to measure dietary intake. Figure 2 provides examples of lunch box photographs and their associated scoring. Where there was ambiguity about whether a lunch box item was an everyday food or a sometimes food (e.g., a grain-based food product that may have been high in sugar) the scorers met to review the item and come to a consensus agreement.

An overall lunch box score was calculated by tallying the number of the five possible food-groups of Everyday foods that were present in a lunch box and subtracting the number of Sometimes foods found. If more Sometimes foods were present than the number of Everyday food groups represented in a lunch box, the score was truncated at zero. Scores for the overall lunch box score ranged from 0 to 5. Everyday food and Sometimes food scores were obtained by simply tallying the number of foods of each type found in a lunch box. There was no maximum value for any food group, but the Everyday food scores ranged from 0 to 12 and Sometimes food scores ranged from 0 to 8. No negative scores were recorded. In Figure 2A, for example, the baseline overall score would be (2-8) = 0 and the post intervention score (2B) is calculated as (5-0) = 5.

### 2.4. Statistical Analysis

Sample characteristics are described in terms of frequencies and percentages for participants’ sex and grade level and for characteristics of participants’ schools, including whether the school was new to the Eat Smart B Active | Healthiest School Awards program, whether the school drew students primarily from a local catchment area, and the decile rank for the Socioeconomic Index for Areas (SEIFA) of the area in which the participant’s school is located.

Means and standard deviations are provided to summarise scores for each dependent variable (Knowledge, Attitudes and three types of lunch box scores: Overall, Sometimes Foods, Everyday Foods) for each of the three occasions on which they were measured. To test the effect of the interventions, separate repeated measures analysis of covariance tests were carried out with each of the respective dependent variables. Time is the condition across which measures of these dependent variables are repeated.

Covariates entered into the analyses to control for the possible confounding influence of different student and school characteristics include sex, student grade level, the school students attended, whether a school was new to the Eat Smart B Active | Healthiest School Awards program, whether or not the school drew students primarily from its local catchment area, and the SEIFA decile of the area in which the school was located.

Interactions between covariates and the dependent variables, and within-subjects effects among covariates, are described and investigated where they are informative. SEIFA decile levels and school location had a close relationship with each other and complex relationships with the dependent variables, often rendering the meaning of interaction effects involving these covariates difficult to interpret. No interpretation of such interactions is presented beyond acknowledging that such interactions show that changes in dependent variables differed across levels of these two covariates.

## 3. Results

In the five schools that participated in the study, there were 215 children in the 10 classrooms selected to participate by school principals. Descriptive characteristics of the study sample (*n* = 212, response rate 98.5%) indicate that participants were evenly divided across sex (girls = 49.1%) with most students in Grades 2 and 4 (82.5%) and a smaller number of students in Grades 3 and 5 (see Table 1). Over three-quarters of the students attended schools that were participating in the program for the first time and almost one-third attended schools that drew their student population from a local catchment area. All students attended schools in areas with relatively low socioeconomic standing with almost half of participants attending a school in the lowest socioeconomic decile.

Table 2 shows the mean score (SD) and range for each dependent variable prior to the start of the program; after the first intervention for the Knowledge and Attitude variables; after the second intervention for all dependent variables; and at the 4–6 week follow-up for the lunch box scores. These data provide the context for the repeated measures analyses and show unadjusted differences in dependent variable scores over time. These results also show that on each testing occasion, children typically had twice as many Everyday foods in their lunch boxes as Sometimes foods.

### 3.1. Change over Time

Tests of differences in the mean scores of the five dependent variables on the three occasions that were each measured are presented below.

#### 3.1.1. Knowledge

Repeated measures analysis of covariance of knowledge scores showed a significant main effect for Time, *F*(1.98, 346.22) = 29.42, *p* < 0.001, after controlling for covariates. This indicates that children’s knowledge improved over the course of the Eat Smart B Active | Healthiest School Awards program. Follow-up tests show that the first intervention (Eat Smart B Active LIVE Show) resulted in a significant increase in knowledge (mean difference = 6.91, SD = 0.30, *p* < 0.001) with a further increase in knowledge (mean difference = 2.63, SD = 0.28, *p* < 0.001) after the second intervention (Two-week lunch box challenge).

**Figure 3:**
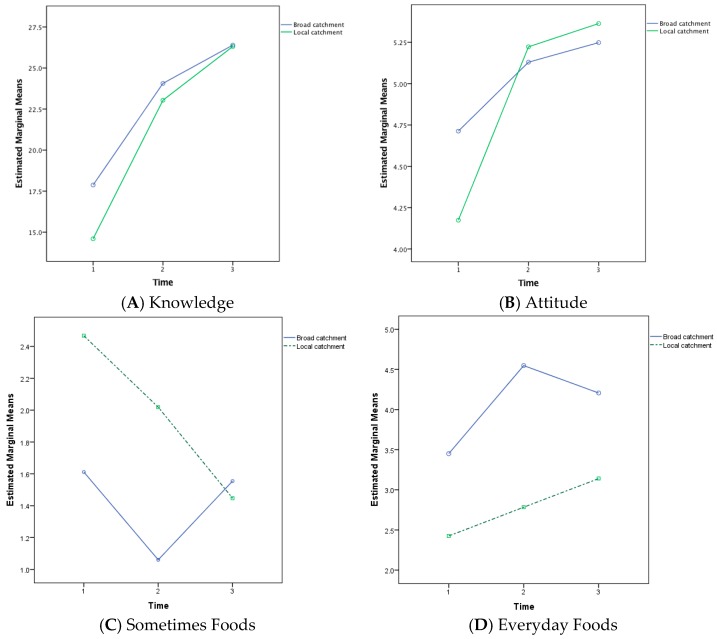
Healthy Eating Interaction Effects by School Catchment for Knowledge, Attitude and Lunch Box Foods (Boxes (**A**,**B**): T1 = baseline, T2 = after theatre show, T3 = after healthy eating competition; Boxes (**C**,**D**): T1 = baseline, T2 = after healthy eating competition, T3 = 4–6 weeks post intervention).

Significant interaction effects were found between knowledge and four of the included covariates; student year level, school new to the program, school catchment and SEIFA decile scores. The student year level interaction was the result of a linear increase in average knowledge scores for Year 3 students in contrast to each of the other grades, which showed a smaller increase in knowledge scores as a result of the second intervention compared to the increase after the first intervention. The catchment area interaction showed that schools drawing students from their local catchment area began with lower average knowledge scores than schools that drew students from beyond their local area but showed more improvement as a result of the interventions (see Figure 3A). Similarly, schools that were new to the Eat Smart B Active | Healthiest School Awards began with lower average knowledge scores than the school that had previously participated in the program but ended the program with higher scores.

#### 3.1.2. Attitude

The multivariate test of difference between the estimated marginal means for Attitude at each time period was significant, *F*(2, 167) = 32.64, *p* < 0.001. Pairwise comparisons showed a significant change in overall attitude scores after the first intervention (mean difference 0.62, *p* < 0.001) but not after the second intervention (mean difference 0.13, *p* = 0.10).

A significant interaction effect was identified between Attitude and school catchment status, *F*(1.96, 328.66) = 3.69, *p* = 0.03. This interaction showed that baseline differences in mean Attitude scores between local and broad catchment area school scores were cancelled out by the first intervention and subsequent increases in attitude were equivalent for both types of school (see Figure 3B). A group difference in Attitude score was also identified between grade levels, *F*(1, 168) = 6.14, *p* = 0.01, with Grade 5 students obtaining lower attitude scores than other grade levels at all three testing occasions.

#### 3.1.3. Lunch Box

Change in lunch box scores over the course of the program are tested across three different indicators. Results are presented below for the overall lunch box score, the score for Sometimes Foods and the score for Everyday Foods.

*Overall Lunch Box Score*. The multivariate test of difference between the estimated marginal means for lunch box score at each testing occasion was significant *F*(2, 161) = 57.59, *p* < 0.001. Pairwise comparisons showed a complex pattern of changes. A significant increase in overall lunch box scores was found between the baseline score and the second test score (after the two interventions: mean difference 1.45, *p* < 0.001). This was followed by a significant decrease in overall lunch box scores at post-test (mean difference −0.85, *p* < 0.001). However, overall lunch box scores at post-test were still significantly higher than at baseline (mean difference 0.59, *p* < 0.001). In addition, a group difference in lunch box score was identified between sexes, *F*(1, 168) = 6.14, *p* = 0.01, with girls scoring higher than boys at each testing occasion.

*Sometimes Foods—Lunch Box Score.* The multivariate test of difference between the estimated marginal means for Sometimes Food score at each testing occasion was significant *F*(2, 161) = 9.85, *p* < 0.001. Pairwise comparisons showed a similar pattern of changes to those found for overall lunch box scores. A significant decrease in Sometimes Foods scores was found between the baseline score and the second test score (after the two interventions: mean difference −0.53, *p* < 0.001). This was followed by a significant increase in Sometimes Food scores at post-test (mean difference 0.22, *p* = 0.031). However, overall lunch box scores at post-test were still significantly lower than at baseline (mean difference −0.31, *p* = 0.015).

A significant interaction effect was identified between Sometimes Food scores and school catchment status, *F*(1.97, 318.43) = 4.84, *p* = 0.009. This interaction indicates that broad catchment area schools showed a drop in Sometimes Foods after the interventions, which recovers to baseline levels at post test. Local catchment area schools, in contrast, showed a steady drop in Sometimes Food scores across all testing occasions (Figure 3C). Group differences in Sometimes Food scores were also identified for school catchment status, *F*(1, 162) = 4.68, *p* = 0.03, with broader catchment area schools obtaining lower Sometimes Food scores than local catchment schools at all three testing occasions; and for sex, *F*(1, 162) = 4.57, *p* = 0.03, with girls having fewer sometimes foods in their lunch boxes than boys at each testing occasion.

*Everyday Foods—Lunch Box Score.* Analysis of Everyday Food scores showed a significant main effect for Time, *F*(2, 324) = 4.84, *p* = 0.018, after controlling for covariates. This indicates that the amount of Everyday Food included in lunch boxes improved over the course of the program. Follow-up tests show that the interventions produced a significant increase in Everyday Food scores at Time 2 (mean difference = 0.91, SE = 0.15, *p* < 0.001) with a small, non-significant drop between Time 2 and the post test (mean difference = 0.16, SE = 0.15, *p* = 0.29).

Group differences in Everyday Food scores were identified for school catchment status, *F*(1, 162) = 13.69, *p* < 0.001, with broader catchment area schools having higher Everyday Food scores than local catchment schools at all three testing occasions (Figure 3D); for sex, *F*(1, 162) = 33.05, *p* = 0.02, with girls having more everyday foods in their lunch boxes than boys at each testing occasion; and for SEIFA, *F*(1, 162) = 81.19, *p* < 0.001, with schools in the two lower decile areas having lower Everyday Food scores than schools in the higher two deciles at each testing occasion.

## 4. Discussion

This study evaluated the impact of the Eat Smart B Active program. As expected, knowledge and attitude scores showed positive change and the improvements were sustained over the short-term.

The results suggest that the bringing together of the child, home and school was effective in improving the quality of the lunch box contents and in enabling healthy eating habits in school children. The doubling of vegetables scores was notable given this is an area of national interest. These results confirm the concept of the “super-setting”, which emphasises the importance of schools’ social and geographical context [9]. This program included two elements that can be considered part of the super-setting. In the first case, the lunch box challenge intervention provided the opportunity for students to take the information they learned home and incorporated it into household lunch box preparation practices. This study also looked at the geographical catchment from which schools drew students.

The program was effective in improving students’ knowledge about healthy eating with separate, significant effects after each program component (theatre production, lunch box challenge). Attitudes towards healthy foods showed less comprehensive improvement, with no effect resulting from the second intervention.

The lunch box scores recorded post-test were lower than the scores collected mid-intervention, however, they were significantly higher than baseline. This demonstrates that children respond to the intervention the most while the program is running in the school. This may be due to the incentive of prizes for eating healthily. However, the post-test scores were still significantly higher than scores taken at baseline, showing that the intervention is effective in developing healthy behaviours that last beyond the period of intervention.

This same trend in adoption of healthy habits was shown in the “sometimes foods” scores. There was a decrease from baseline to the second test, then an increase of “sometimes foods” from the second to the post-test. Overall, the post-test score was significantly lower than the baseline score. However, the analysis of “everyday foods” scores demonstrated increasing improvement throughout the duration of intervention and into post-test. Thus, while it seems “sometimes foods” were starting to be re-introduced into lunch boxes after the program, the understanding of “everyday foods” where consistently evident throughout the course of the study period. 

Across the grade levels participating in the study, students in the 5th year level were found to have lower attitude scores than any of the other grade levels over all three tests, indicating the possible increased difficulty of changing nutritional behaviours as children get older [19,20,21]. Girls also tended to demonstrate better outcomes of behaviour change than boys; girls recorded higher lunch box scores than boys on every occasion (lower “sometimes foods” scores, and higher “everyday foods” scores [22].

The geographical catchment from which the schools were, also contributed varying outcomes to the program. Results showed a consistent interaction effect where students from schools that drew from a local catchment area started with lower scores on each of the dependent variables measured (knowledge, attitude and lunch box contents) but showed larger improvements over the course of the program than students at schools drawing from a broader catchment area. Interestingly, analysis of “sometimes foods” results across catchment areas, showed that broader catchment area schools tended to show a drop during the intervention and then a return to baseline in the post-test, whereas local catchment area schools showed a stead decrease in “sometimes foods” across all three measurement occasions.

Taken as a whole, the results from this study indicate that theatre production can act as a dynamic motivator and an effective educational tool, particularly in combination with a competitive challenge. A strength of this study and an important feature of this program is that the foods brought to school by students over the course of the program and again a month later were evaluated directly, rather than relying on student self-report. This also means that there was very little loss-to-follow-up for post-test results. However, while the program has been effective in enabling short-term, behaviour changes around healthy eating habits, the one-month follow-up meant that it was not possible to determine the longer-term sustainability of outcomes of the program. 

School and student sampling is another limitation of this study. It is possible that more motivated principals elected to take part in the study and that they chose classes that were more likely to benefit from the intervention to participate. Those factors could limit the effects of the intervention with less motivated school communities.

As a population-based intervention, there was no collection of anthropometric or biochemical data as this was felt to not only be invasive but would likely diminish participation. It is, therefore, a limitation that no correlations could be made between body composition and behaviour.

While there was no formal evaluation of study variable score reliability, the method of data collection was strong in limiting bias. The use by researchers of a standard scoring protocol for photographed lunch box content increased confidence in behavior scores. This study would have benefitted from the inclusion of control schools, however the use of each school as its own control over time allows some conclusions to be drawn about the value of this particular form of intervention. This is supported by including and adjusting for six potential confounding school and student-level factors.

Parental understanding on healthy eating can also significantly determine the learned nutritional behaviours of their children [23,24,25]. Future program implementation may also consider the involvement of and feedback from parents in the intervention to enhance potential for the longevity of the outcomes of the program, and to maximise the effects of the “super-setting” theory.

## 5. Conclusions

The Eat Smart B Active program has been shown to be an effective and integrative approach to improving school children’s nutritional knowledge, attitude, and behaviours. Despite the limitations of the study, the findings of this study provide strong support for the effectiveness and success of using theatre performance as an educational tool, alongside the incentive of a challenge, to provide school children with the knowledge, attitude and behaviours of eating healthy, in the short-term. The study has supported the notion of “super-setting”, and highlights the value and success of a multifaceted approach to behaviour change. Further research is required to determine the longevity of the program outcomes. 

## Figures and Tables

**Table 1:** Descriptive characteristics of participating students.

Sample Characteristics	Number (%)
Sex	
Female	104 (49.1)
Male	108 (50.9)
Grade Level	
Grade 2 (ages 7–8)	70 (33.0)
Grade 3 (ages 8–9)	17 (8.0)
Grade 4 (ages 9–10)	105 (49.5)
Grade 5 (ages 10–11)	20 (9.4)
New to Program	
Yes	165 (77.8)
No	47 (22.2)
Local Catchment School	
Yes	64 (30.2)
No	148 (69.8)
SEIFA Decile	
1	102 (48.1)
3	27 (12.7)
4	46 (21.7)
5	37 (17.5)
Total N	212

SEIFA: Socioeconomic Index for Areas

**Table 2:** Mean scores (SD) and range of scores for knowledge, attitude and lunch box challenge at three time periods.

Variable	Baseline	After Show	After Lunch Box Challenge	Post Test
Knowledge Score	16.83 (6.3)	23.74 (5.8)	26.37 (4.5)	
	Range 28 (2–30)	Range 26 (4–30)	Range 26 (4–30)	
Attitude Scale Score	4.54 (1.3)	5.16 (1.1)	5.31 (1.1)	
	Range 5 (1–6)	Range 5 (1-6)	Range 6 (0–6)	
Overall Lunch Box Score	1.28 (1.4)		2.73 (1.6)	1.88 (1.5)
	Range 5 (0–5)		Range 5 (0–5)	Range 5 (0–5)
Sometimes Foods	1.83 (1.5)		1.31 (1.2)	1.53 (1.5)
	Range 8 (0–8)		Range 11 (0–11)	Range 7 (0–7)
Everyday Foods	3.18 (1.7)		4.10 (1.9)	3.93 (2.0)
	Range 8 (0–8)		Range 8 (0–8)	Range 12 (0–12)

## References

[1] Mbizvo E. (2006). Essay theatre—A force for health promotion. Lancet.

[2] Joronen K., Rankin S.H., Astedt-Kurki P. (2008). School-based drama interventions in health promotion for children and adolescents: Systematic review. J. Adv. Nurs..

[3] Lobstein T., Baur L., Uauy R. (2004). Obesity in children and young people: A crisis in public health. Obes. Rev..

[4] World Health Organisation (WHO) (2016). Report of the Commission on Ending Childhood Obesity.

[5] Commonwealth of Australia (2007). Australian National Children’s Nutrition and Physical Activity Survey—Main Findings.

[6] Australian Bureau of Statistics (2014). National Nutrition Survey Undertaken in 2011–12.

[7] Lee A. (2009). Health promoting schools: Evidence for a holistic approach to promoting health and improving health literacy. Appl. Health Econ. Health Policy.

[8] Department of Health (2014). Food and Nutrition Policy 1992.

[9] Bloch P., Toft U., Reinbach H.C., Clausen L.T., Mikkelsen B.E., Poulsen K., Jensen B.B. (2014). Revitalizing the setting approach—Supersettings for sustainable impact in community health promotion. Int. J. Behav. Nutr. Phys. Act..

[10] Glanz K., Rimer B.K., Lewis F.M. (2002). Health Behaviour and Health Education: Theory, Research and Practice.

[11] Carter M.A., Swinburn B. (2004). Measuring the ‘obesogenic’ food environment in new zealand primary schools. Health Promot. Int..

[12] Gasevic D., Vukmirovich I., Yusuf S., Teo K., Chow C., Dagenais G., Lear S.A. (2011). A direct assessment of “obesogenic” built environments: Challenges and recommendations. J. Environ. Public Health.

[13] Scaglioni S., Salvioni M., Galimberti C. (2008). Influence of parental attitudes in the development of children eating behaviour. Br. J. Nutr..

[14] Cheadle A., Cahill C., Schwartz P.M., Edmiston J., Johnson S., Davis L., Robbins C. (2012). Engaging youth in learning about healthful eating and active living: An evaluation of educational theatre programs. J. Nutr. Educ. Behav..

[15] Perry C.L., Zauner M., Oakes J.M., Taylor G., Bishop D.B. (2002). Evaluation of a theater production about eating behavior of children. J. School Health.

[16] Frederick-Recascino C.M., Schuster-Smith H. (2003). Competition and intrinsic motivation: A comparison of two groups. J. Sport Behav..

[17] Johannesson M., Östling R., Ranehill E. (2010). The effect of competition on physical activity: A randomized trial. B.E. J. Econ. Anal. Policy.

[18] Queensland Government Schools Directory. https://schoolsdirectory.eq.edu.au.

[19] Ashcroft J., Semmler C., Carnell S., van Jaarsveld C.H.M., Wardle J. (2008). Continuity and stability of eating behaviour traits in children. Eur. J. Clin. Nutr..

[20] Davis M.M., Gance-Cleveland B., Hassink S., Johnson R., Paradis G., Resnicow K. (2007). Recommendations for prevention of childhood obesity. Pediatrics.

[21] Liu Y.H., Stein M.T. (2013). Feeding behaviour of infants and young children and its impact on child psychosocial and emotional development. Encyclopedia on Early Childhood Development.

[22] Lin W., Yang H.-C., Hang C.-M., Pan W.-H. (2007). Nutrition knowledge, attitude, and behavior of taiwanese elementary school children. Asia Pac. J. Clin. Nutr..

[23] Golley R.K., Hendrie G.A., Slater A., Corsini N. (2011). Interventions that involve parents to improve children’s weight-related nutrition intake and activity patterns—What nutrition and activity targets and behaviour change techniques are associated with intervention effectiveness?. Obes. Rev..

[24] Gruber K.J., Haldeman L.A. (2009). Using the family to combat childhood and adult obesity. Prev. Chronic Dis..

[25] Rhee K. (2008). Childhood overweight and the relationship between parent behaviors, parenting style, and family functioning. Ann. Am. Acad. Political Soc. Sci..

